# *DIMScern*: A Framework for Discerning *DIMSE* Services on Remote Medical Devices

**DOI:** 10.3390/s24237470

**Published:** 2024-11-22

**Authors:** Gunhee Kim, Dohyun Kim, Jeonghun Seo, Seyoung Lee, Wonjun Song

**Affiliations:** 1 Department of Convergence Security, Kangwon National University, 1 Kangwondaehak-gil, Chuncheon-si 24341, Republic of Korea; gunheekim@kangwon.ac.kr (G.K.);; 2 Department of Computer and Information Engineering, Catholic University of Pusan, Busan 46252, Republic of Korea; 3 Division of Cardiology, Department of Internal Medicine, Kangwon National University Hospital, Baengnyeong-ro 156, Chuncheon-si 24289, Republic of Korea; 4 Department of Computer Science and Engineering, Kangwon National University, 1 Kangwondaehak-gil, Chuncheon-si 24341, Republic of Korea

**Keywords:** *DICOM*, *DIMSE*, biomedical imaging, security

## Abstract

In the medical domain, computer systems in digital healthcare have increased connectivity continuously and the *DICOM* Message Service Element (DIMSE) protocol has a critical role in exchanging biomedical imaging data among different digital healthcare systems. As the data communication technology is used to handle sensitive information such as patient information (e.g., patient’s name, date of birth, and address) and medical images (e.g., ultrasound, X-ray, and MRI), it has emerged as a major target for security attacks. In this work, we study security concerns on the message exchange method used in the *DIMSE* protocol. It is important to know which *DIMSE* services are available on a given healthcare IT system to an adversary and we observe that the *DIMSE* protocol can be implemented in various ways across products, with each supporting different *DIMSE* services as well. We present *DIMScern*, a framework for discerning *DIMSE* services on remote medical devices. To show the effectiveness of *DIMScern*, we evaluate our framework on multiple *DIMSE* implementations, including commercial products and libraries, and identify the supported *DIMSE* services of them. We demonstrate that *DIMScern* successfully identifies medical services that are supported differently across 22 healthcare IT systems in a remote environment.

## 1. Introduction

Healthcare IT systems, encompassing both software and hardware, are increasingly being deployed in various healthcare environments and are progressively transitioning to online connectivity. Recently, security concerns surrounding healthcare IT systems have intensified due to their increasing reliance on online connectivity. Remote connectivity broadens the attack surface on the digital healthcare systems, providing adversaries with increased opportunities for malicious activities such as information leakage, malware, sensor-spoofing, and data corruption attacks. Prior work [1] reported data breaches of 385 M patients’ records and showed an annual increase in security threats related to data leakage [2,3]. They analyzed that the information leakage predominantly occurs within network environments. Medical sensors have been a major target as they are used to measure physiological vital signs from patients. Previous research demonstrated a security attack targeting the malicious control of an infrared (IR) drop sensor by saturating the sensor [4], alongside a data corruption attack aimed at compromising the integrity of a CT device [5]. Additionally, various studies have conducted analyses on the security threats (e.g., ransomware, phishing, and denial-of-service attacks) associated with the healthcare IT environment [6,7,8].

However, collecting information on the target and accurately identifying the target system are fundamental initial steps in the aforementioned diverse security threats. Previous studies proposed several methods for probing the target systems in network environments [9,10,11,12,13,14] across various security domains.

In this work, we demonstrate how information on healthcare IT systems can be remotely accessed and analyzed, including patient information (e.g., name and biomedical imaging data), sensor name, and S/W version. In particular, we also explain the method for determining what types of medical services are provided by a remote system.

In the past, healthcare IT systems used different file formats and different data transmission methods for each manufacturer, and as this diversity increased, a single integrated healthcare IT system protocol emerged, which is the *DICOM* (Digital Imaging Communications in Medicine) protocol [15,16,17]. In particular, it explains how healthcare services and medical data are exchanged and structured in packet units for network communication between healthcare IT systems. To probe healthcare IT systems that can be accessed online, we present a framework to identify medical services that are supported by the target system. And we show that our framework can also be used to obtain patients’ personal information and details of the target system, including S/W version, product name, and sensor name, which are exploited to enable different security attacks based on its corresponding CVE (Common Vulnerabilities and Exposures) [18,19,20,21,22].

We evaluate the performance of our framework on 22 *DIMSE* implementations from 11 vendors and show that our framework correctly identifies medical services (or *DIMSE* services) for all experimental targets. In particular, the main contributions of our work include the following.

- We investigated several implementations, including commercial medical devices, libraries, and open-source frameworks of the *DIMSE* protocol.
- We observed differences between the *DIMSE* specification and its implementation and exploited them to identify medical services on the remote systems.
- We propose a software framework, *DIMScern*, which automatically sends a crafted packet and parses its response packet to discern all SOP (Service-Object Pair) Class UIDs that are supported by a target imaging device.
- We demonstrate how the *DIMScern* framework is exploited to leak sensitive information including patients’ data and sensor identifiers from remote systems.

The source code of our framework can be publicly accessed, and the experimental settings and results used in this work are available at https://github.com/CASO-Lab/DIMScern.git (accessed on 22 October 2024).

## 2. Background

### 2.1. *DICOM* and *DIMSE* Protocols

A variety of methods have been used to exchange data between healthcare systems, and each system uses different file formats for healthcare data. To unify these methods, the *DICOM* (Digital Imaging and Communication in Medicine) protocol was developed in 1985 as a medical image processing standard by the ACR (American College of Radiology) and NEMA (National Electrical Manufacturers Association) to facilitate compatibility with various healthcare IT systems. The reason the *DICOM* protocol was created was that in the past, each manufacturer had different storage methods and data exchange methods in the medical system. As this phenomenon increased, a unified method was needed, and so *DICOM* standard was created.

**Figure 1:**
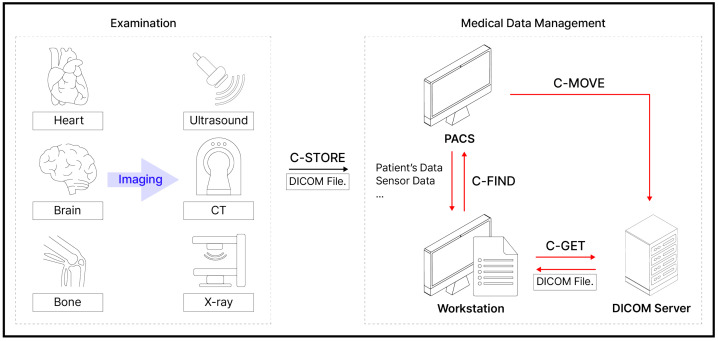
Overview of *DICOM* and *DIMSE* operations among healthcare IT systems.

*DICOM* is a broad standard that covers the entire system for storing, transmitting, searching, and viewing medical images. *DICOM* specifies the file format, transmission protocol, and data format of image data. Of these, the *DIMSE* protocol is part of *DICOM* and is the protocol responsible for transmission. *DIMSE* (DICOM Message Service Element) is a protocol that indicates the method of exchanging data between entities using a network. An overview of *DICOM* and *DIMSE* in the overall healthcare system is shown in Figure 1.

### 2.2. DICOM File

Various healthcare IT systems measure and store data about a patient’s physical condition or health status using different types of sensors. These data come in various forms, such as images and text. In the DICOM protocol, a patient’s imaging data and related information are integrated and stored together in a single file format.

Addressing the need for standardization across diverse systems, a standardized method for storing data—the medical data storage file format—was introduced. This file format defines how medical data, including images and associated metadata, is organized and stored, enabling an efficient and unified format for data exchange across diverse medical systems.

**Figure 2:**
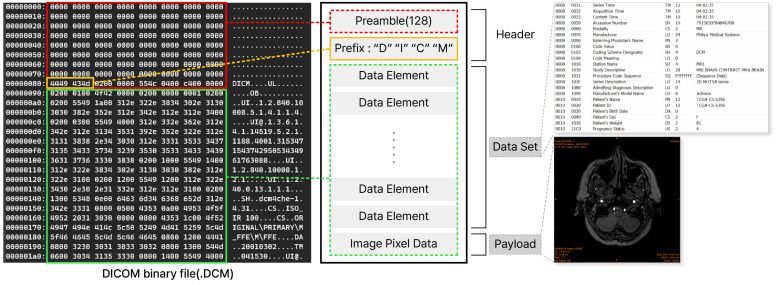
The DICOM file format structure ( in this figure, we used a DICOM file from *The Cancer Imaging Archive (Creative Commons Attribution 3.0 Unported License)* [23], in which *Data Set* and *Payload* are integrated).

The file used in the *DICOM* protocol is a DICOM file, and the extension is usually “.dcm”. This file is created by taking a specific patient image or video and making it into a single file and includes patient information, shooting information, equipment information, etc. The DICOM file format is largely divided into two parts: the *Header* part and the *Data Set* part, as shown in Figure 2.

The *Header* part usually has a *Preamble* part filled with 128 bytes of “0”. This Preamble exists for applications that do not recognize the DICOM file, and in such cases, the image offset is filled in this part, and the application uses the offset to process the image directly. Next, there is the Prefix part, which contains the strings “D”, “I”, “C”, and “M” encoded to indicate the DICOM file format. This value is a fixed value, and all DICOM files must contain the corresponding value in the prefix part.

The *Data Set* part contains various *Data Elements* and *Image Pixel Data*, and the *Data Elements* store metadata through tags defined in *DICOM*. There are several types of such metadata, such as patient information, medical equipment information, and examination information. Patient information includes (0010, 0010) patient name, (0010, 0030) patient birth date, (0010, 1040) patient address, etc. Equipment-related information includes (0008, 0070) manufacturer, (0018, 1020) software version, (0014, 3022) sensor name, etc. Information related to captured examination results includes (0008, 0020) examination date, (0008, 0030) examination time, etc. Image-related information includes (0008, 0008) image type, (0028, 0008) frame count, etc. This way, not only the patient’s examination images, but also the patient’s personal information, examination results, equipment information, and image information are all included in a single DICOM file.

In the following subsection, we introduce how these DICOM files are exchanged between healthcare IT systems in the *DIMSE* protocol.

### 2.3. Network Communication in Healthcare IT Systems via *DIMSE*

Many healthcare IT systems utilize networks to share medical data. These medical data include not only DICOM files but also specific medical services. For example, various services exist, such as remotely storing medical data, requesting the transfer of medical data, or querying specific medical data. The *DIMSE* protocol defines the entities involved in transmitting and receiving medical data between healthcare IT systems. In this subsection, we explain the stages of the exchange process between entities and the types of medical services available. Through this, we provide insights into the operational principles of medical data exchange between healthcare IT systems using the *DIMSE* protocol.

There are two main entities in a *DIMSE* relationship: SCU and SCP. SCU stands for “Service Class User”, which corresponds to the client-side role, and SCP stands for “Service Class Provider”, which corresponds to the server-side role. SCU is the entity that initiates the message exchange and requests information or operations from SCP. Conversely, when SCP receives information from SCU, it passes that information to SCU or processes it. In the context of SCU and SCP, various entities are involved, including medical devices, Workstations for displaying medical images or videos for diagnosis, PACS (Picture Archiving and Communication System) for storing and managing such data, and *DICOM* servers for searching, transmitting, and storing medical images. These are not limited to the role of one entity.

**Figure 3:**
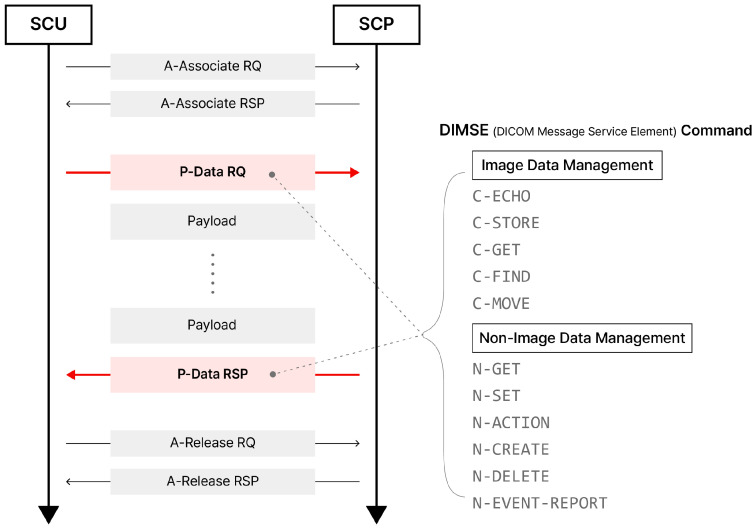
Packet exchange process of *DIMSE* protocol between entities.

In the *DIMSE* protocol, the exchange of medical data or services between these two entities is designed to be performed step by step. These steps, as shown in Figure 3, are divided into three main phases: A-Associate, P-Data, and A-Release.

A-Associate is a step for establishing a connection between entities, which involves request and response procedures. The response can result in two outcomes: acceptance or rejection. Only when this step is successfully completed can the process proceed to the next step, P-Data. P-Data is the stage where DICOM files or data related to specific medical services are exchanged. These data are exchanged between entities through specific commands in the *DIMSE* protocol. Once the data exchange between the two entities is complete, the connection is terminated in the A-Release stage, where the connectivity between the two entities is released and finalized.

**Figure 4:**
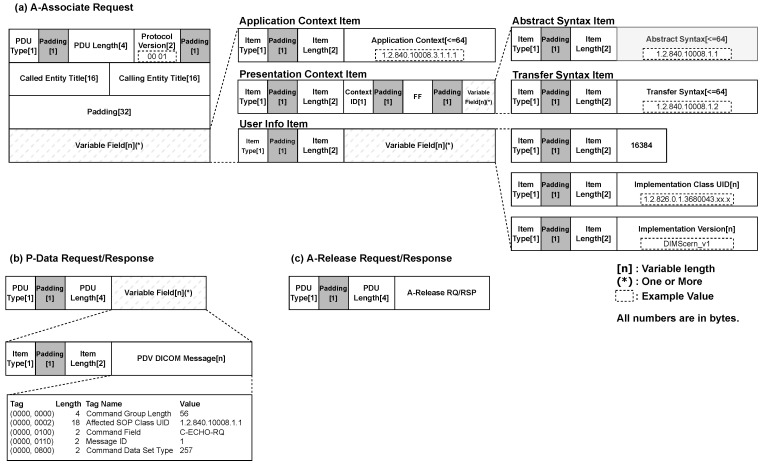
Field structure of *DIMSE* packet.

A high-level overview of the actual data flow in each stage of the *DIMSE* protocol is shown in Figure 4, and we will explain the components used in each stage. In the following Section 2.3.1, we examine what data are exchanged when establishing connections between healthcare IT systems, and in Section 2.3.2, we explore what medical services are requested and executed.

#### 2.3.1. Managing Medical Services and Healthcare IT Devices in *DIMSE*

Healthcare IT systems integrate and manage devices and medical services to facilitate data exchange. We examine how these elements are integrated and managed, as well as what types of medical data are exchanged.

The *DIMSE* protocol introduces the concept of the *SOP (Service-Object Pair) Class*, which combines medical services and healthcare IT devices. This serves as a fundamental structure for managing the transmission and processing of medical data, consisting of a pair of a service and an object. *SOP Class* is a critical component when transmitting data or performing operations in the *DIMSE* protocol. Services include functions such as storage, query, retrieval, and verification, while objects encompass types like MRI, CT, X-ray, and Ultrasound. For example, when storing medical data generated by an ultrasound device—specifically a DICOM file—on a *DICOM* server, the “Ultrasound Image Storage” *SOP Class* is employed. This *SOP Class* is defined within the *DIMSE* protocol to facilitate such operations.

Requesting a medical service from one healthcare IT system to another requires transmitting information about the *SOP Class*, which allows the *DIMSE* protocol to determine what services are available for a specific system. For this information to be transmitted, a connection process between the two entities is required, which is defined as the A-Associate phase in the *DIMSE* protocol. As shown in Figure 3, this is the very first stage of the data exchange process. The packet fields used to establish this connection are shown in Figure 4a. These fields consist of multiple components, and their purposes are outlined in Table 1.

The *Variable Field*, unlike other previous fields, has a variable length. This is because the length of its subfields varies depending on the medical service, requiring a unified variable-length structure. The *Variable Field* is broadly divided into three categories: *Application Context Item*, *Presentation Context Item*, and *User Info Item*.

1. Application Context Item: This item consists of the *Application Context Name* field and its item length; currently, only “1.2.840.10008.3.1.1.1” is allowed in the *Application Context Name* field in *DIMSE*.
2. Presentation Context Item: This Item is largely composed of two items and their length fields.
  - *Abstract Syntax Item*: This item has an *Abstract Syntax* field which contains the *SOP Class UID*. For example, the *SOP Class UID* for storing CT images is “1.2.840.10008.5.1.4.1.1.2”. To store CT images, we need to send this UID as a packet and receive a response. The corresponding *SOP Class UID*s are listed in *DICOM* Standard.
  - *Transfer Syntax Item*: This item has a *Transfer Syntax* field, which contains the UID for the byte order and compression method that represents the *DICOM* encoding rules. For example, the *Encoding representing Implicit VR Endian*: Default Transfer Syntax for *DIMSE* would have the value “1.2.840.10008.1.2”.
3. User Info Item: This item consists of the maximum PDU length field, the Implementation UID, and the *Implementation Version* field. One example is 16384, “1.2.826.0.1.36800 43.xx.x”, “DIMScern_v1”.

We have explained the first stage of exchanging information about medical services, which is the A-Associate stage, and described the essential packet fields. Once the A-Associate stage is completed, the connection between the two healthcare IT systems is established, and in the next stage, medical data related to medical services are exchanged, or specific tasks are requested and performed.

#### 2.3.2. Data Exchange and Operation Notification in Digital Healthcare IT Systems via *DIMSE*

Once the connection between healthcare IT systems is established, medical data and operation notifications related to medical services are practically exchanged. This stage is defined as the P-Data phase in the *DIMSE* protocol, which, as shown in Figure 3, corresponds to the second phase. In this stage, data are exchanged using specific commands. For example, when one healthcare IT system intends to store a specific DICOM file on another healthcare IT system, one of the commands defined in the *DIMSE* protocol is transmitted through a packet during the P-Data phase. The requested storage operation is then performed, and the result is exchanged. This section explains the commands used for exchanging medical data and the packet fields used in this stage.

The commands used for exchanging medical data between healthcare IT systems are divided into two types: DIMSE-C and DIMSE-N.

DIMSE-C (Composite) is a message exchange method for exchanging requests between composite SOP instances. There are five types of commands (or queries), such as C-ECHO, C-STORE, C-GET, C-FIND, and C-MOVE.

- C-ECHO: This command is used to check the connection between entities. For example, the SCU sends a C-ECHO command to the SCP before sending medical data, to verify that the connection with the SCP is stable.
- C-STORE: This command is used by the SCU to store a DICOM file from its local storage to the SCP. For example, when attempting to store a DICOM file from a medical device to a PACS or a server, the SCU (medical device) sends a C-STORE command to the SCP (PACS or *DICOM* server) and stores the DICOM file on the SCP.
- C-GET: This command is used when the SCU wants to obtain a DICOM file that exists on the SCP. For example, if the SCU requests a DICOM file matching a specific patient’s name using the C-GET command, the SCP sends the corresponding DICOM file to the SCU.
- C-FIND: This command is used when the SCU wants to obtain data from a DICOM file that matches specific criteria. For example, if the SCU sends a C-FIND command to the SCP to retrieve the gender of a patient from a DICOM file with the patient’s name “CASO”, the SCP sends the corresponding patient gender data to the SCU.
- C-MOVE: This command is used by the SCU to store a DICOM file from the SCP to a third storage location. For example, the SCU sends a C-MOVE command to the SCP to store a DICOM file with the patient name “CASO” from the SCP (PACS) to another SCP (*DICOM* server).

DIMSE-N (Normalized) is a notification and service that can be applied to a normalized SOP instance. There are six types of commands (or queries), such as N-GET, N-SET, N-ACTION, N-CREATE, N-DELETE, and N-EVENT-REPORT.

- N-GET: This command is used when the SCU requests specific information from the SCP. For example, the SCU can use the N-GET command to request information about a print job from the SCP, and the SCP responds to the SCU with the result of the operation.
- N-SET: This command is used by the SCU to modify the attributes of specific information on the SCP. For example, the SCU can use the N-SET command to change the number of copies for a print job on the SCP. The SCP then updates the task accordingly and sends the SCU a response with the result of the operation.
- N-ACTION: This command is used by the SCU to request the SCP to perform a specific task. For example, the SCU can use the N-ACTION command to request the SCP to start a print job. The SCP executes the print job and then sends the SCU a response with the result of the operation.
- N-CREATE: This command is used by the SCU to request the SCP to create a specific task. For example, the SCU can use the N-CREATE command to create a print job. The SCP generates the print job object as requested and sends the SCU a response with the result of the operation.
- N-DELETE: This command is used by the SCU to request the SCP to delete a specific task. For example, the SCU can use the N-DELETE command to request the deletion of a print job. The SCP deletes the corresponding object for the task and sends the SCU a response with the result of the operation.
- N-EVENT-REPORT: This command is used by the SCP to notify the SCU about events such as task status updates or error occurrences. For example, the SCP sends an N-EVENT-REPORT command to the SCU regarding the occurred event, and the SCU receives the event and sends an acknowledgment response back to the SCP.

These commands are contained in packets and transmitted during the P-Data stage, as shown in Figure 4b. The purpose of these fields is outlined in Table 2.

The *Variable Field* field has a variable length and contains the actual *DIMSE* command within its subfields.

- *PDV DICOM message*: The PDV (Presentation Data Values) contains the contents of the *DIMSE* command. For better understanding, an example of the value contained in this field when the C-ECHO command is executed is provided and is shown in Table 3.
  In this example, the values for the C-ECHO command are located within specific tags in the *PDV DICOM message field*.

Once medical data are exchanged between healthcare IT systems using *DIMSE* commands, the connection between entities is terminated in the A-Release phase. This represents the final phase in Figure 3, and the packet fields for this phase are shown in Figure 4c. A-Release is a step of disconnecting when there are no more data to exchange after exchanging data between individuals. After this step, the connection between entities is completely disconnected.

### 2.4. Related Work

Numerous studies have explored research topics related to software technologies on sensor data and its data communication within healthcare IT systems and they are largely divided into three categories: studies related to the implementation of the *DIMSE* protocol, studies on the security aspects of the *DIMSE* protocol, and studies on the security aspects of DICOM files. We briefly introduce each of them and their relevance to our research topic.

***DIMSE*** **protocol implementation.** Campos et al. [24] classified and explained internal commands related to *DIMSE* according to the *DICOM* service. In addition, they created a framework called Medical Imaging Workflows Analyzer and presented two sensors. Among these, the framework showed the *DIMSE* protocol using a network sensor and presented the detailed structure. This study appropriately paired the commands of the *SOP Class* and the *DIMSE* protocol, and through this, we found out the relationship between the *SOP Class* and *DIMSE* command when designing the framework.

Mantri et al. [25] introduced and reviewed *DICOM* libraries. *DICOM* developers consider which library to choose from, and this is an important decision. The libraries implementing the *DICOM* protocol found on the Internet, in theses, and on Github are listed, with each characteristic compared. This study represents whether each library is open-source, which OSs are supported, how much SOP support is given, and what languages are available for development. Through this, it is expected that each library can be selected according to their characteristics, advantages and disadvantages, and the developer’s needs. It helped us collect targets for the *DICOM* Library/Toolkit when designing and evaluating our upcoming framework.

Vidyashree et al. [26] describe the implementation of the *DIMSE* protocol in detail. Their study describes the commands belonging to *DIMSE*, shows the message structure and primitives in the actual *DIMSE* protocol, and describes the message fields and parameters of C-ECHO, C-STORE, and C-MOVE in detail. It also explains the relationship between DICOM files and the *DIMSE* protocol. It describes the A-Associate request, the steps to accept the request, and the response steps of C-ECHO, C-STORE, C-FIND, and C-MOVE in P-Data in detail through log capture. This study details the message fields for C-STORE, C-FIND, and C-MOVE of the actual *DIMSE* protocol through three stages, which helps in understanding the A-Associate stage, which is the most core part of our framework design.

***DIMSE*** **protocol security.** Mileva et al. [27] present the relationship between the *DIMSE* protocol and covert channel generation. Their study explains the P-Data phase of the *DIMSE* protocol using C-ECHO as an example and presents the methodologies for creating covert channels according to each *DIMSE* command. It presents a scenario for covert channel generation using entropy and presents the results of experiments and evaluations. What can be learned from this study is that *DIMSE* is not only a message exchange method, but can also create covert channels related to security. The merit of this study is that it presents a methodology for creating a secret channel for each command of the *DIMSE* protocol, and it shows through simulation whether a secret channel is possible by creating an actual experimental scenario. Through this study, we provide a view that the packets of the *DIMSE* protocol can be viewed from a security perspective.

Karagiannis et al. [28] use *DICOM* libraries and Docker to enhance the understanding of *DIMSE* protocols, propose a possible cyberattack method, and build a simulation environment for this. In the *DICOM* protocol, data exchange has a client and a server subject, which explains the communication process and process between two entities. In addition, the *DICOM* libraries “pydicom” and “pynetdicom” are used to act as the client subject, and “Orthanc” is configured as the server subject in the virtual environment. After writing several codes to the client, an environment in which the server can communicate through the *DICOM* protocol is configured. Since then, it is said that it has provided an environment that can simulate cyberattacks. This study creates a simulation environment that implements the *DIMSE* protocol and provides an environment for security testing by building this environment. Through this, we have figured out a methodology for building a *DIMSE* protocol environment, and through this, we have learned the overall structure of the *DIMSE* protocol, which has helped us design our framework.

**DICOM file security.** B. Desjardins et al. [29] cover *DICOM* security and present research on it. There are two main attacks on the *DICOM* protocol: data access and data injection. First, for data access attacks, the Massachusetts Institute of Technology found about 2700 unprotected *DICOM* servers on the Internet, and McAfee researchers used Shodan [30] to find about 1100 unprotected *DICOM* servers worldwide. This allowed them to obtain all the patients’ data. The second is data injection attacks. Security researchers have introduced a method to inject or remove data from patient image data using deep learning techniques sophisticated enough to fool radiologists. This study may have different results in that it can change *DICOM* images. Another data injection attack is a technique to hide malware in DICOM files. In the DICOM file section, if we put the data of the DOS header in the “Preamble” and put the malware in the “Data Element”, when the victim where the DICOM file is located opens the file, the malware will be executed and the system will be damaged. Through this paper, we learned that the *DIMSE* protocol is connected with *DICOM* security, and we were able to design our framework to contribute to the *DIMSE* security aspect in the future.

Mileva et al. [31] introduce a secret channel generation method that can occur in terms of information concealment through the DICOM file steganography technique. The text length of the “Data elements” of *DICOM* should be defined as even, and if it is odd, it is automatically padded to an even length. In this case, secret data are created here by taking advantage of the fact that there is no limit to the padding byte. In the above study, 0.5-bit, 1-bit, 4-bit, and 8-bit secret data were inserted into each DICOM file, and the recipient received them through experiments. The study proposed a method to generate secret channels using steganography techniques and stated that the solution to this is to calculate the blank character ratio or to check the *DICOM* input. This study presented an information hiding channel methodology through the characteristics of DICOM files, and through this, we found that the command to the C-STORE that transmits the DICOM file in our framework is also important in terms of security.

Subhasri et al. [32] suggested a way to protect DICOM files. It is to encrypt the DICOM file using a “Visener password”. The DICOM file has a large image pixel part and a data part, *Data Element* part, and the tags corresponding to that image are contained in the *Data Element* part. This tag also contains the patient’s personal information. In this study, the DICOM file is converted into a BMP file, and then the image pixel part and the tag part are encrypted through the “Visener password”. It revealed the process of encryption and decryption, and as a result of measuring the actual encrypted DICOM file in terms of performance, it indicated that encryption was successful and the image quality was excellent. This study presented a methodology to safely encrypt patient data or equipment information in DICOM files and exchange secure files through this.

## 3. Security Concerns on *DIMSE*

In the previous sections, we focused on the characteristics of protocols designed for managing biomedical imaging data and facilitating network communication among healthcare IT systems. However, security flaws often are arise from the discrepancy betweens a protocol’s specification and its implementation. Thus, we tried to analyze two types of medical systems, a commercial *hardware* product and an open-source *software* where the *DIMSE* protocol is implemented for medical services, in order to observe their security concerns.

**Figure 5:**
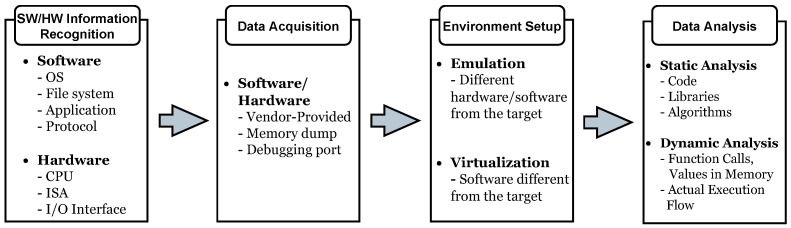
Overview of reverse-engineering procedure.

Reverse-engineering is a commonly used technique to analyze security concerns in a specific system and we first outline the general procedure and the necessary components required for this technique in Figure 5.

The entire procedure of reverse-engineering can be broadly divided into four stages: *Software/Hardware Information Recognition*, *Data Acquisition*, *Environment Setup*, and *Data Analysis*.

The first stage, *Software/Hardware Information Recognition*, involves pre-emptively identifying and cataloging information about the target before performing the analysis. This preparatory step is crucial for planning and facilitating subsequent stages effectively. In this stage, software-related information such as the operating system (OS), file system, application details, and protocols is identified. For hardware, details such as the CPU model, instruction set architecture (ISA), and types of I/O interfaces are gathered.

The next stage is *Data Acquisition*, which follows the completion of information collection about the target. This stage focuses on obtaining data from the target system using various methods. A common approach involves vendors providing data about specific hardware or software systems, typically in the form of documentations, datasheets, or similar resources. Another widely used technique for data acquisition is the memory dump method, which involves directly extracting firmware or software from a device’s memory through interfaces such as serial ports or other memory access methods. *Data Acquisition* through the debugging port method is a further approach, enabling direct access to the firmware, software, or other data of the target system via debugging interfaces without the need for memory dumps. For instance, connecting to UART or other debugging ports allows accessing and extracting the target’s software and other data.

After obtaining the software or binaries of a specific system, setting up an environment for runtime analysis becomes essential. This process involves replicating the target system’s environment on a separate system for analytical purposes. Such an environment is necessary when the analyst’s setup differs from that of the target system, ensuring compatibility and offering the flexibility to employ various analysis methods as needed. There are two primary techniques for achieving this: emulation and virtualization. Emulation is a method that reproduces the behavior of the target system’s hardware or software on another system. It enables the execution of binaries from the target on entirely different platforms by translating the instruction set architecture (ISA) of the target processor. Virtualization involves creating a virtual version of the target system within a different environment. Unlike emulation, virtualization is primarily used when the host system’s hardware is compatible with the requirements of the target.

After configuring the environment to suit the analysis needs, the process proceeds to the *Data Analysis* stage. At this stage, the target system can be analyzed within a controlled runtime environment tailored to the analyst’s preferences. This stage is divided into two main categories: static analysis and dynamic analysis. Static analysis involves examining the target without executing the software. This includes analyzing code at a high-level language, assembly, or binary level. Key areas of focus during this phase include reviewing code, identifying libraries used by the target, and understanding algorithms of interest to the analyst. Dynamic analysis, on the other hand, involves executing the software and analyzing it in a runtime environment. During this phase, the primary focus is on observing function calls, values in memory, and the actual execution flow of the software. These static and dynamic analysis steps constitute the final stage of the reverse-engineering process.

We conducted reverse-engineering on various healthcare IT systems (*Vivid-i* [33], *DCMTK* [34], Merative [35], Leadtools [36], dcm4che [37], and pynetdicom [38]) based on the steps described above, categorizing them into two representative types: software and hardware. Using a case study approach, we present the methods and results, including detailed procedures and findings, to provide a comprehensive understanding of the system structures and potential risks associated with healthcare IT systems.

### 3.1. Case Study of Medical Hardware System

In this subsection, we present an in-depth analysis of one of our hardware-based healthcare IT systems, *Vivid-i* [33]. *Vivid-i* is a portable medical device that uses *ultrasound* to monitor cardiac events from patients. The device enables medical technicians (e.g., doctors and clinical engineers) to capture diagnostic information and saves the measured data as an image or video for further analysis.

#### 3.1.1. Hardware System Information Recognition and Data Acquisition

**Figure 6:**
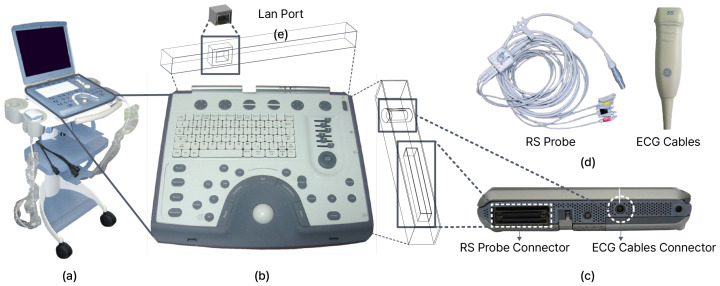
(**a**) The complete structure of *Vivid-i*; (**b**) keyboard and operator panel; (**c**) ports for probe and cables; (**d**) RS probe and ECG cables; (**e**) LAN port. ( Elements of this figure are referenced from [33,39,40]).

The major components of the medical device are shown in Figure 6. *Vivid-i* (Figure 6a) has several interfaces, among which the ones related to *DIMSE* are introduced. The *Keyboard and Operator Panel* (Figure 6b) has a keyboard and buttons for various functions, among which the “Store” button has the function of exchanging *DIMSE* data with the outside. On the right side (Figure 6c) are ports related to sensors, including the *ECG Cable Connector and RS Probe Connector*. These are for connecting the *ECG Cables* and RS Probe. The *ECG Cables* is used to monitor and record the electrical signals of the heart, and the *RS Probe* uses an ultrasound sensor to contact the actual patient’s body to examine the patient’s internal condition and collect data (Figure 6d). On the back, there is a LAN port for entering and exiting *DIMSE* data with the outside (Figure 6e). The overall process is to collect patient medical data through sensors (Figure 6d) connected to the port (Figure 6c), save it as a DICOM file in the medical device, and communicate it with the outside world through a button (Figure 6b), and such communication is achieved through a LAN port (Figure 6e).

**Figure 7:**
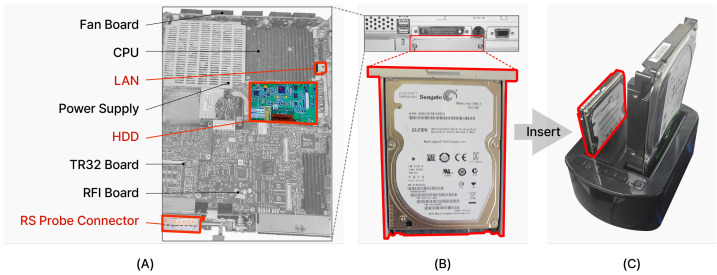
The hardware of *Vivid-i* (**A**) and HDD clone process (**B**,**C**).

The medical device has an Intel Pentium M processor CPU, Windows XP Embedded-Service Pack 2 operating system, and 2GB RAM. The hardware image of the medical device is shown in Figure 7A, and we have marked the *CPU*, *Power Supply*, *RFI Board*, *TR32 Board*, *Fan Board*, LAN, and *RS Probe Connector*. The *RS probe connector* is located in the same position as the RS probe in Figure 6c and is connected to an ultrasonic probe that includes an ultrasonic sensor. The function of the ultrasonic sensor is to transmit ultrasonic waves inside the body, detect the reflected sound waves, and convert them into images. In addition, since it uses radiation that is harmless to the human body, safe examination is possible. *Vivid-i* measures the patients’ body condition through the *RS probe* equipped with this ultrasonic sensor.

We found out through hardware reversing that the medical device has an HDD (Hard Disk Drive) as a secondary storage device (Figure 7B). This HDD is a 500 GB Seagate HDD, partitioned into four logical drives, and we confirmed that all of *Vivid-i*’s data are stored in this HDD.

*Vivid-i* features include C-ECHO and C-STORE for SCP. C-ECHO can check if a connection is possible with the configured SCP, and this can be achieved by going into the configuration screen in the software. C-STORE can transfer DICOM files inside the medical device to the configured SCP and this can be achieved using a specific button on the medical device. These functions interact with external systems, and through packet capture, we discovered that they utilize the *DIMSE* protocol.

#### 3.1.2. Environment Setup and Data Analysis

**Figure 8:**
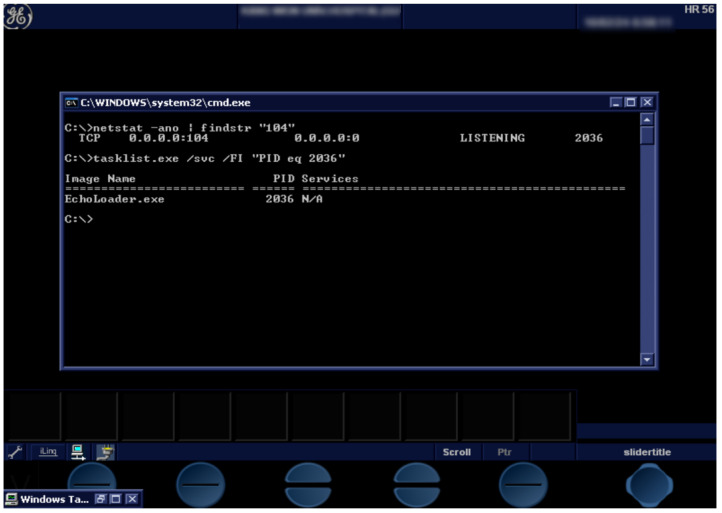
*Vivid-i* healthcare IT system boot screen after rehosting in virtual machine environment.

We went through the process of creating a system identical to the original in a new environment to analyze the relationship between *Vivid-i* and the *DIMSE* protocol. We cloned the medical device HDD to a new HDD via the docking station using the 500 GB *Seagate* HDD (Hard Disk Drive) built into the *Vivid-i* (Figure 7B,C). After that, we converted the new HDD into a virtual hard disk file using Disk2vhd [41] to make it a single disk image in the software. To build a new environment, the local system was built on Intel^®^ i5-1035G7 @ 1.20 GHz, 8 GB RAM and Windows 10 64bit. Since the medical device, ISA is x86 and the local system ISA is x86_64, *Oracle VirtualBox* [42] was used as a virtualization platform because it is compatible at the machine language level. The virtual hard disk file was converted to a bootable file in *VirtualBox*, VDI (VirtualBox Disk Image), and the environment for system booting was successfully created as shown in the result screen of Figure 8. We confirmed that the same environment was configured by comparing it to the actual medical device, and we modified the batch file used for system booting in the rehosted environment to enable the use of *Task Manager* and *Command Prompt*. After checking the open processes in the system through the *Command Prompt*, we found the PID of the process using port 104 in the TCP protocol, and when we searched for the process name of this PID through the *Command Prompt*, we found that it was EchoLoader.exe. By checking the CPU usage of the process through Task Manager and terminating the process, we confirmed that EchoLoader.exe is the main program of *Vivid-i*.

We created an environment identical to the medical device in a new environment, so any analysis was possible for our purposes. Our interest was whether it was possible to execute the *DIMSE* command from the outside to a medical device, and we performed a simple test to actually check this. Using *DCMTK* [34], an open-source library that uses the *DIMSE* protocol, we generated packets and transmitted them to the medical device. Except for C-ECHO among *DIMSE* commands, the remaining *DIMSE* commands all received a rejection response from the medical device.

We analyzed why only C-ECHO could be transmitted to medical devices from the outside. First, we analyzed the main program using the dynamic analysis debugger x32dbg [43] and Cheat Engine [44]. After sending the packet, we set a breakpoint at the point where the packet was stored and looked for where this buffer was referenced. Through analysis, we found that this buffer was used by a dynamic library called by the main program. As a result of analyzing this dynamic library, we found that the task of processing *DIMSE* packets over the network was performed in this library.

**Figure 9:**
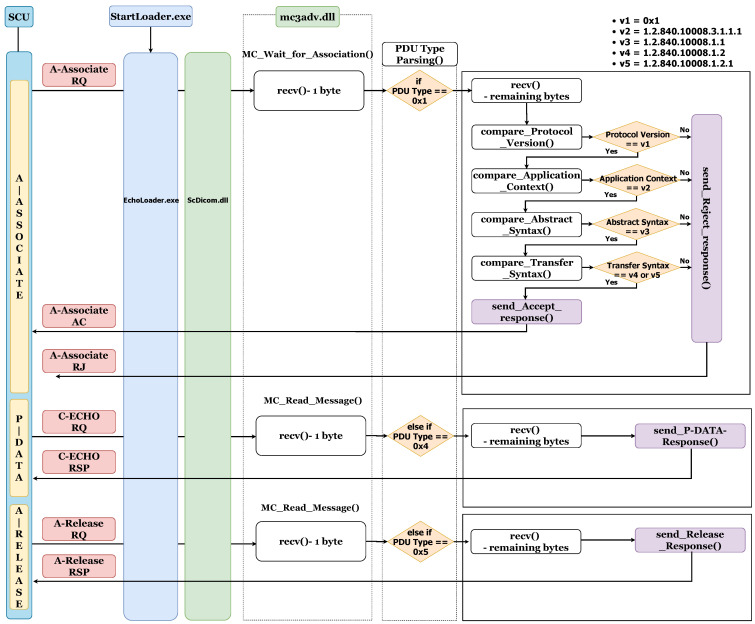
Software structure of *Vivid-i* through static and dynamic analysis.

The software structure of *Vivid-i* is as shown in Figure 9. *StartLoader.exe* is executed when *Vivid-i* boots up, and it is a process that restarts *EchoLoader.exe* when it is terminated. *EchoLoader.exe* is the main program of this medical device system. All tasks start with this program. *MergeCOM-3* is a toolkit for integrating and managing *DICOM* tasks and supports many dlls and settings. Among them, *mc3adv.dll* is a library that processes *DIMSE* and contains several functions. We will explain the flow focusing on the function that processes *DIMSE* packets among several functions. When an A-Associate request packet comes from the SCU, it waits in the listen state in the MC_Wait_for_Association function and receives 1 byte first. The execution flow is determined by the *PDU Type*, which is the first byte of the *DIMSE* packet. Since the *PDU Type* of A-Associate request value is 0 x1, the control flow receives the remaining bytes, and then checks the *Protocol Version* field, *Application Context* field, *Abstract Syntax* field, and *Transfer Syntax* field. If any of them do not match, *Vivid-i* sends an A-Associate rejection packet to SCU, and if all of them are satisfied, it sends an A-Associate acceptance packet to SCU. SCU sends the *SOP Class UID* for C-ECHO to *Vivid-i*, and *Vivid-i* assumes that it has accepted the *SOP Class UID* and moves on to the next step, the P-Data step. Now in P-Data, SCU sends a C-ECHO request packet to *Vivid-i*. Then, *Vivid-i* reads 1 byte through the MC_Read_Message function, and since that 1 byte is 0 x4, which is the value for P-Data, it proceeds to the flow for 0 x1 in the *PDU Type Parsing* function. Then, it generates a C-ECHO response packet and sends it to SCU. In the final step, A-Release, when SCU sends an A-Release request packet to *Vivid-i*, it reads one byte through the MC_Read_Message function, and since that one byte is 0 x5, which is the value of P-Data, the *PDU Type Parsing* function proceeds the flow for 0 x5. Then, by generating an A-Release response packet and sending it to the SCU, the two entities are separated from the *DIMSE* protocol.

We confirmed that commands other than C-ECHO were not sent from outside to *Vivid-i*. To find the cause, we will explain why other *DIMSE* commands except C-ECHO are not possible through static and dynamic analysis of the library. We performed static analysis on the dynamic library through *IDA Pro* [45] and performed dynamic analysis with x32dbg at the same time. We organized the functions that process packets first, receive, and transmit, and assumed that there was a reason why the *DIMSE* command, not C-ECHO, was rejected at the midpoint of receiving and transmitting packets, and analyzed the relationship between them.

In order to do that, we need to send C-ECHO and another *DIMSE* command, and for this analysis, we passed the C-STORE to this environment. During the analysis, we found a point where the return values of a specific function were different when performing C-ECHO and C-STORE, and after analyzing the cause, we found a part where the values of a specific packet field are compared. The algorithm that expresses this part is in Algorithm 1, and analyzing it in detail, it determines the return value by comparing the value of the “Abstract Syntax” field of the A-Associate request packet with the value already set in the medical device. The already set value is “1.2.840.10008.1.1”, which can be found in the *SOP Class UID* defined in the *DICOM* standard. This UID is defined as “Verification Class” and is used when performing the C-ECHO operation in *DIMSE*. If the value of the *Abstract Syntax* field in the request packet is “1.2.840.10008.1.1”, line 10 returns 1, and if it is not “1.2.840.10008.1.1”, line 12 returns 0. Based on the return value of this compare_Abstract_Syntax function, the behavior of the upper function changes to determine A-Associate rejection (return 0) and A-Associate acceptance (return 1).
**Algorithm 1** *SOP class UID* comparison pseudo-code  1:**compare_Abstract_Syntax** (int arg1,int arg2, DWORD *arg3) {  2:   …  3:   char buffer_A[size_a];  4:   char buffer_B[size_b];  5:   …  6:   buffer_A = * (*SOP Class UID* in *Abstract Syntax* field from A-Associate request)  7:   buffer_B = "1.2.840.10008.1.1"  8:   …  9:   if (strcmp (buffer_A, buffer_B) == 0)10:      return 1; //Accept11:   else12:      return 0; //Reject13:}

### 3.2. Case Study of Medical Software System

We analyzed several pieces of medical software (*DCMTK* [34], Merative [35], Leadtools [36], dcm4che [37], and pynetdicom [38]), but in this subsection, we present an in-depth analysis of the implementation of *DCMTK*, a specific software product provided as open-source.

#### 3.2.1. Software System Information Recognition and Data Acquisition

*DCMTK* [34] is an open-source project that implements the *DIMSE* protocol. It includes APIs, libraries, and test programs. There are various types of libraries, and they include various functions such as data encoding/decoding, compression/decompression, networking, image database, and image processing. Our point of analysis among these is related to the networking part, which is a library that implements the *DIMSE* protocol. We found test programs that use libraries that implement the *DIMSE* protocol, and among them, we analyze *storescp.exe*, which is an SCP role that supports C-STORE. *DCMTK*, being open-source, allows for static analysis at the source code level, and the source code for the dynamic library *dcmnet.dll*, used in the application program, is also publicly available. Unlike the hardware systems described in Section 3.1.1, data acquisition for the target system is relatively straightforward, as it can be accomplished online by downloading the code provided by the developer. Since the data for the target system have been acquired, the process proceeds to the environment setup and data analysis stages for further analysis.

#### 3.2.2. Data Analysis in Software System

**Figure 10:**
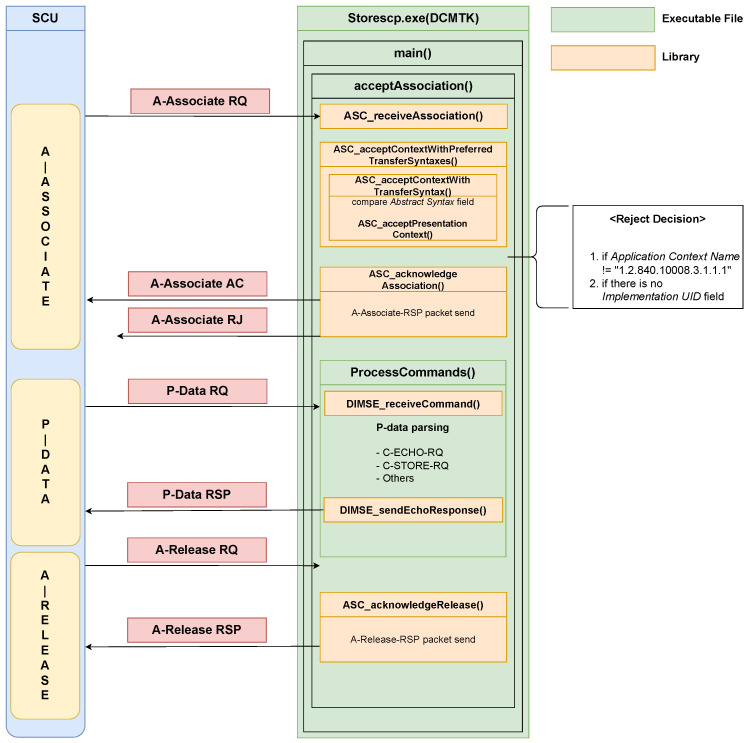
Software structure of *DCMTK (storescp.exe)* through static and dynamic analysis.

*DCMTK* provides test programs that can be used on Windows, and among them, *storescp.exe*, a program that uses the *DIMSE* protocol of our interest, can be analyzed without having to build a new runtime environment in our experimental environment. We performed static analysis on the code provided as open-source and dynamic analysis while sending and receiving actual *DIMSE* packets. Through our analysis, we present the flow of *DIMSE* packet processing in *storescp.exe* of *DCMTK* and the software structure of the corresponding test program, as shown in Figure 10.

When an A-Associate request packet arrives from SCU, *storescp.exe* processes it in the acceptAssociation () function of the main function. There are several functions inside, and the place where the packet is actually received is a function in the dynamic library dmcnet.dll. The ASC_receiveAssocaition function receives the packet, and the next function is the ASC_acceptContextWithPreferredTransferSyntaxes () function, which parses the packet fields. This function parses the *Abstract Syntax* field of the *Presentation Context* field among the packet fields to indicate whether the corresponding *SOP Class UID* is supported.

After that, acceptance or rejection is determined, and the A-Associate response packet is sent to the SCU. If acceptance is sent when the SCU sends a packet requesting data along with a command in the P-Data phase, *storescp.exe* parses the command in the DIMSE_receiveCommand () function inside the ProcessCommands () function. C-ECHO, C-STORE, and others are parsed, and the flow changes depending on which command it is. In our analysis, C-ECHO was sent as a command, and *storescp.exe* proceeded with the flow for C-ECHO. The P-Data response packet is sent to the SCU through the DIMSE_sendEchoResponse () function. After that, the SCU sends the A-Release request packet to *storescp.exe*, and the ASC_acknowledgeRelease () function of *dcmnet.dll* receives the packet and sends the A-Release response packet to the SCU and the connection between the two entities is terminated.

An important point in the analysis of *storescp.exe* is that when deciding between acceptance and rejection at the A-Associate stage, it is largely divided into two categories:

- If the *Application Context Name* is not “1.2.840.10008.3.1.1.1”;
- If the *Implementation UID* field is empty.

Outside of these conditions, no rejection is determined, i.e., even if the *SOP Class UID* is not supported by *storescp.exe*, the acceptance packet is sent to the SCU.

### 3.3. Observations and Conclusions

In Section 3.1, we presented a detailed analysis process and results of how the hardware system in healthcare IT systems processes medical services and medical data using the *DIMSE* protocol. Similarly, in Section 3.2, we presented a detailed analysis process and results of how the software system in healthcare IT systems processes medical services and medical data using the *DIMSE* protocol.

The common observations obtained from the case studies of hardware and software systems in various healthcare IT systems are as follows:

- We discovered that the *SOP Class UID*s are pre-defined differently for each of targets because the supported medical services vary on the medical systems.
- There is a file that defines a list of *SOP Class UID*s for each platform, but we found that only some of the lists in the file are used depending on the application characteristics, even on the same platform.
- We found that it is possible to determine which medical services the medical IT system supports through remote packet analysis.

In Section 3.1 and Section 3.2, through the analysis of *Vivid-i* and *DCMTK*, we observed that the value in the *Abstract Syntax* field of the A-Associate request packet, specifically the *SOP Class UID*, is compared with the target’s pre-defined *SOP Class UID*s to determine whether the response is acceptance or rejection. This behavior is further explained through additional experiments by sending *SOP Class UID*s that are not pre-defined by the targets.

**Figure 11:**
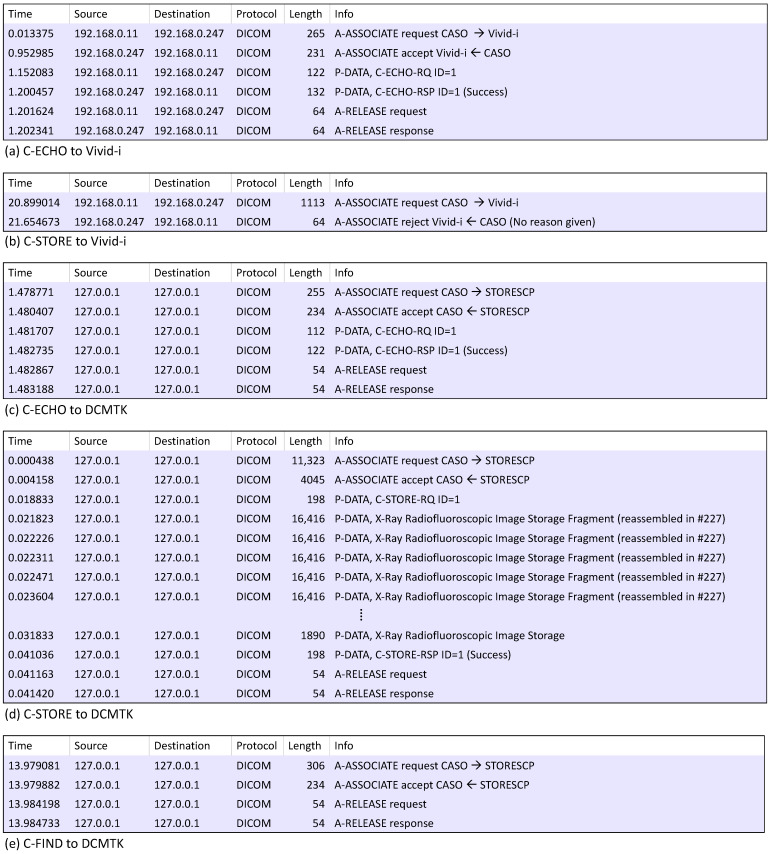
*DIMSE* packet capture from *Vivid-i* and *DCMTK*.

The packet exchange process involved executing C-ECHO and C-STORE commands on *Vivid-i* and capturing the packets. Additionally, C-ECHO, C-STORE, and C-FIND commands were performed using *DCMTK*’s *storescp.exe*. During this process, network packets were captured using the Wireshark [46], and the results are presented in Figure 11.

In Figure 11, Figure 11a,b are *DIMSE* processing packet captures in *Vivid-i*, a medical device, and Figure 11c–e are *DIMSE* processing packet captures in *storescp.exe* of *DCMTK*. Figure 11a shows that SCU performed C-ECHO on *Vivid-i*, and A-Associate request, response, C-ECHO request, response, A-Release request, and response were performed. Each step was performed normally, and C-ECHO was performed normally in the *DIMSE* protocol, because the *Verification Class* “1.2.840.10008.1.1” in the Abstract Syntax of the A-Associate request is the *SOP Class UID* pre-defined by *Vivid-i*. Figure 11b shows that SCU performed C-STORE to *Vivid-i*, and included a UID other than Verification Class UID of A-Associate request, in this case, *Vivid-i* responds with an A-Associate rejection packet because the *SOP Class UID* is not pre-defined. Figure 11c shows that SCU performed C-ECHO to *storescp.exe*, and A-Associate request, response, C-ECHO request, response, A-Release request, and response were performed. Each step was performed normally, and like Figure 11a, C-ECHO was performed normally in the *DIMSE* protocol because the *Abstract Syntax* of the A-Associate request includes the *SOP Class UID* for the *Verification Class* “1.2.840.10008.1.1”, which is pre-defined by *storescp.exe*. Figure 11d shows that SCU performed C-STORE on *storescp.exe*, and A-Associate request, response, C-STORE request, and response, A-Release request, response were performed. This is because when *storescp.exe* inspected and compared the *Abstract Syntax* field, it showed a normal packet flow because the *SOP Class UID* used for C-STORE is also pre-defined by *storescp.exe*. Figure 11e shows that SCU performed C-FIND on *storescp.exe*, and as a result, A-Associate response, response, and A-Release request, response were performed. This is because the *SOP Class UID* for C-FIND is not pre-defined by *storescp.exe*.

In the algorithms identified through the analysis of each target, examining the part where the *Abstract Syntax* field is inspected and compared reveals that in the case of *Vivid-i*, only the *SOP Class UID* for C-ECHO is accepted, while all other *SOP Class UID*s are rejected. *DCMTK* is only the *SOP Class UID*s for C-ECHO and C-STORE, and the remaining *SOP Class UID*s are rejected. When the two targets compare the *Abstract Syntax* field of the A-Associate request packet, it refers to a subset of the *SOP Class UID* list defined in the configuration file or header file. *Vivid-i* defines the *SOP Class UID*s in “mergecom.srv” and *DCMTK* defines the *SOP Class UID*s in "dcuid.h", and what we observed is that only some of the *SOP Class UID*s in this list are used depending on the needs of the application. In addition to the two case studies, we observed that other systems (Merative [35], Leadtools [36], dcm4che [37], and pynetdicom [38]) similarly read a list of *SOP Class UID*s from a specific file, adopt a part of *SOP Class UID*s in the list to specify medical services that are supported by the system, and then compare them with the *SOP Class UID*s in the request packet to determine the response.

Through the analysis of various healthcare IT systems, we observed an additional point: it is possible to remotely identify the medical services that can be performed by a specific healthcare IT system using packets. In practice, actual *DIMSE* commands are executed during the P-Data phase, while the associated *SOP Class UID*s are exchanged during the A-Associate phase. Therefore, even before reaching the P-Data phase, the A-Associate phase allows for determining which *DIMSE* commands, and consequently which medical services, are available based on the *SOP Class UID*. This eliminates the need to verify the pre-defined *SOP Class UID* list for each target or perform reverse-engineering. Instead, this information can be obtained remotely by sending packets to the target and analyzing the responses.

## 4. Design of *DIMScern*

We conducted case studies on different healthcare IT systems in Section 3 and observed that an *SOP Class UID* has its corresponding medical service and an *SOP Class UID* is directly related to a particular *DIMSE* command as well. This section introduces the design for discerning *DIMSE* commands in remote medical IT systems by leveraging these characteristics. Particularly, we introduce a framework, *DIMScern*, which can determine medical services that are supported on the remote target system and our framework can possibly be used to leak sensitive data such as patient information, manufacturer, and sensor name. We begin by describing the design considerations of our framework, followed by presenting its high-level algorithm.

### 4.1. Design Consideration

Based on our analysis in the previous section, the A-Associate step verifies an *SOP Class UID* in a received packet and see if its corresponding medical service is available on the healthcare IT systems. Specifically, an SCP can find the value of an *SOP Class UID* in the *Abstract Syntax* field of the A-Associate packet. The SCP sends an accepted packet back to the SCU if it supports the *SOP Class UID* or a rejected packet if it does not support it. However, we observed that *storescp.exe* of *DCMTK* did not send a rejection packet for the *SOP Class UID* (e.g., *C-FIND*) that is not supported in the software.

Rather, we identify the unsupported *SOP Class UID* from the SCP by checking its response packet containing the value “*03*”, meaning *Unsupported Abstract Syntax* in the *Presentation Context Result* field.

We found out that even if the value of the *PDU Type* field has acceptance (0 x00), we need to check the detailed field, the *Presentation Context Result* field. Therefore, the framework we designed at first identified only the *PDU Type* of the A-Associate response packet, but in the case of *storescp.exe* of *DCMTK*, we could not identify it only by *PDU Type*, and we needed to check the *Presentation Context Result* field as well, so we created a framework that identifies it by considering this field.

### 4.2. Design Strategy

We expected that if we included all *SOP Class UID*s defined in *DICOM* in the A-Associate request packet and passed them to the target using the *DIMSE* protocol, we could distinguish between acceptance and rejection through the response. This would allow us to know which *SOP Class UID*s are accepted at the A-Associate stage and which *DIMSE* commands are possible for the target based on those UIDs without going to the P-Data stage.

We collected all *SOP Class UID*s defined according to the *DICOM* standard documents [47,48]. This resulted in 257 *SOP Class UID*s, which have been listed. When designing a method to include these UIDs when sending packets to the target, we decided to provide one UID as an input value for the packet field. After sending a request packet to the target, if a response is received and the target supports it, acceptance is the result, so we used a method to record only the *SOP Class UID* accepted according to this criterion in a single output file.

A packet must be generated through input (a list of *SOP Class UID*s), and in this part, we designed a method to generate packets by observing the packet generation methods of various toolkits and finding rules.

The algorithm of the framework we designed is as Algorithm 2. For a target using the *DIMSE* protocol, we create an A-Associate request packet by inserting one *SOP Class UID* at a time, sending this, and then receiving a response. We determine whether the target supports the *SOP Class UID* by looking at the *PDU Type* field, which is the first byte of the response packet. Through analysis, we found that we need to additionally check and determine not only the *PDU Type* field, but also the *Presentation Context Result* field in the response packet, so we added line 13.
**Algorithm 2** *DIMScern* pseudo-code1:**def main ():**2:3:   for uid in sop_class_uid_list ():4:5:      #Packet Generate6:      request = A-Associate_request (uid)7:      send (request)8:9:      recv (response)10:11:      #Discern12:      if response[0,1] == "01": #PDU Type field13:         **if response[210,211] == "00":** #Added Presentation Context Result Field14:         record_output_file (uid)15:      else:16:         continue17:}

We designed the fixed parts of the packet structure of *DIMSE* so that users can change the parts that can be changed. For example, when sending a packet to a target, we set default values for *Calling AE Title* and *Called AE Title* and allowed users to send packets by entering desired values in the corresponding fields as needed.

These defaults are the ones we set for fields that are used as fixed values in the *DIMSE* protocol and for fields that are not user-defined, and are shown in Table 4.

This not only does not require code modification but is also useful when users want to send packets by changing the values of specific fields for the target. And the method of receiving a response packet and checking whether the corresponding *SOP class UID* is accepted by the target and is designed to parse specific fields according to the acceptance and rejection criteria of the *DICOM* standard and record the acceptance result.

Using this method, we designed a logic to send a packet containing all *SOP Class UID*s of the A-Associate request packets one by one and receive a response to it. Through this framework, we sent the packet to *Vivid-i* and captured the packet with Wireshark [46] at the same time. As a result of comparing the frameworks that record packets and responses, *Vivid-i* only accepts *Verification Class UID* and does not accept the remaining *SOP Class UID*. That means that it only accepts C-ECHO among the *DIMSE* protocol. We explained the reason why it accepts only C-ECHO in the algorithm earlier, and it means that the logic and design methodology are appropriate.

What we achieved in previous Section 3 is that if we want to know the *SOP Class UID* supported by the target and the available *DIMSE* commands, we can perform static analysis on *DCMTK* since it is open-source and, if necessary, perform dynamic analysis to find the *Abstract Syntax* comparison part through reverse-engineering. In the case of *Vivid-i*, since the application program is just a binary, we can only perform dynamic analysis. Also, to reiterate what we mentioned at the end of Section 3.3, although there are multiple lists of *SOP Class UID*s used by the target in a specific file, not all of them are used when applied in an actual application-level program, so it is not possible to know which *SOP Class UID* the target supports by looking at the list file. Since the time required to find out which *DIMSE* commands are available for the target is long and requires a complex level of analysis by applying the above method, we designed a framework to remotely identify the *SOP Class UID* supported by the target and the available *DIMSE* commands in a short time through packets.

We created a framework that can apply this method to any target that uses the *DIMSE* protocol, not just medical devices. If we apply the above methodology to a target that uses the *DIMSE* protocol, we can find out which *DIMSE* commands are possible (e.g., C-ECHO, C-STORE, C-FIND, C-GET, C-MOVE, etc.). The methodology we have developed so far is only for one medical device, and we need to design it in detail so that it can be applied to any target that uses the *DIMSE* protocol. To do so, we need to pay attention to the values of the fields corresponding to the reasons for rejection in the response among the A-Associate fields. We have processed all of those values and made it possible to change the values of the fields that should be different for each target. The detailed implementation of our framework is introduced in the next section.

## 5. *DIMScern* Framework Implementation

**Figure 12:**
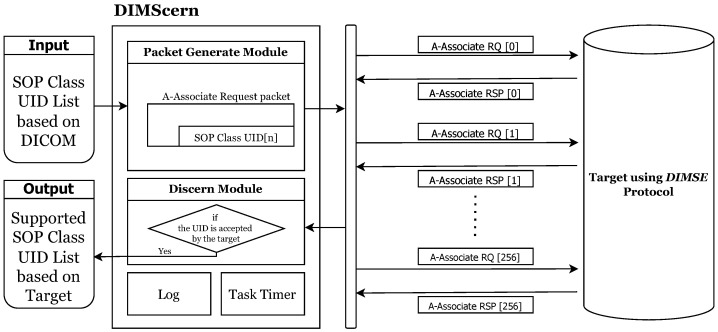
*DIMScern* framework overview.

This section introduces the framework implementation. The purpose of the framework is to classify the *SOP Class UID* allowed for a target using the *DIMSE* protocol, and based on the results, the user can decide the possible *DIMSE* command for the target. The framework, called *DIMScern*, is implemented in Python and consists of two main modules and two functions, as shown in Figure 12.

The input of *DIMSCern* is an *SOP Class UID* list containing all *SOP Class UID*s defined in the *DICOM* standard.

**Packet generation module.** The packet generation module generates an A-Associate request packet in the *DICOM* connection process. The generation process places the *SOP Class UID*s in the input list (*SOP Class UID* list) one by one in the *Abstract Syntax* field and fills in the values for the remaining fields. Since the *Protocol Version* and *Application Context* have fixed values, they are filled in with fixed values, and the *Called Entity Title* and *Calling Entity Title* fields are configured to be changeable because the values accepted by each target are different.

**Discerning module.** After sending packets to the target through the Packet generation module, we receive packets for A-Associate response and determine whether the target accepts the corresponding *SOP Class UID*. If it accepts it, we record the *SOP Class UID*, and if it does not, we do not record it.

The framework output records only the *SOP Class UID* allowed by the specific target.

There are two functions separate from the module. The first function is the Log function, which logs the number of *SOP Class UID*s allowed by the target and the time the framework was used. The second function is the Task Timer, which measures the period from when the first A-Associate request packet is sent until the 257th A-Associate response is received.

**Examples of Framework Flows.** We explain the overall process with an example, assuming that our framework is applied to a specific target. Once the target is determined, *DIMScern* first retrieves the first *SOP Class UID* from the input file and includes it in the A-Associate request packet, which is then sent to the target. After that, the target sends back an A-Associate response packet, which will either be an acceptance or a rejection. We only log to the output file if it is an acceptance; otherwise, it is not recorded. This process is repeated for a total of 257 *SOP Class UID*s. Once all UIDs have been sent and responses received, the system logs the time taken and the number of *SOP Class UID*s accepted by the target. After the process is complete, the output file contains only the *SOP Class UID*s that were accepted by the target, providing insight into which *DIMSE* commands can be used to communicate with the target.

## 6. Evaluation

To conduct an evaluation considering diverse healthcare IT systems, we selected 22 experimental targets using the *DIMSE* protocol from 11 vendors, as shown in Table 5. We performed our experiments on an Intel® i5-1035G7@ 1.20 GHz, 8 GB RAM, Windows 10 64 bit [49]. The targets shown in Table 5 can be classified into three groups: medical devices, libraries/toolkits, and PACS/servers.

**Medical device:** Healthcare IT devices are deployed in the medical site and they are used to measure and examine patients. In particular, the medical devices that have physical sensors such as X-ray, CT, and ultrasound produce medical imaging data which can be combined with patients’ information and converted into a DICOM file. The *DIMSE* protocol is used to control medical devices and to transmit the medical imaging data. In this work, we evaluate the effectiveness of our framework on a particular implementation of the *DIMSE* protocol in a commercial medical device, *Vivid-i* [33].

***DICOM*** **Library/Toolkit:** The *DICOM* library/toolkit is designed to comply with the *DICOM* standard and provides developers with a variety of APIs and source code in multiple programming languages for easy integration. Many *DICOM* libraries and toolkits are available online, but we focused on those that support the *DIMSE* protocol. The target vendors we collected were Merative [35], Leadtools [36], *DCMTK* [34], dcm4che [37], and pynetdicom [38]. We set the *DIMSE* communication-enabled test program provided by these vendors as our target.

***DICOM*** **PACS/Server:** In the medical field, PACS (Picture Archiving and Communication System) is an integrated system for storing, managing, retrieving, transmitting, and viewing medical data. It primarily stores medical images digitally and supports efficient access to medical data in real-world healthcare systems. A *DICOM* server is used for searching, transmitting, and storing medical data, with a focus on communicating with other medical systems. As this group of target, we evaluated Orthanc [50], SonicDICOM [51], Sante [52], Dicoogle [53], and ConQuest [54].

We assume that *DIMScern* is connected to the same network as an experimental target and also assume that the IP address and port of the target is known in advance; to utilize our framework, the target healthcare IT system, whether hardware-based or software-based, simply needs to be connected to the same network. For instance, a security analyst can install our framework, *DIMScern*, on their personal computer and execute it. Once *DIMScern* is running, it can identify the available medical services on the target system and discern which commands can be executed on the target system as a result. In addition to the network IP and port at the A-Associate stage, we observed that some targets require additional information, *Calling AE (Application Entity) Title* and *Called AE (Application Entity) Title*, which enable valid communication on the network.

We note that we also observed that the requirements vary depending on the target’s implementations, so we classified the targets into four types as below:

**Type I. Target that only accepts specific** ***Called AE Title***

In our experimental setup, the target acts as the SCP, so the Called AE Title refers to the AE Title of the target. If the Called AE Title in the request does not match the target’s *AE Title*, the target will not accept the request. When the *Called AE Title* in the request packet does not match, the request is rejected.

**Type II. Target that only accepts specific** ***Calling AE Title***

The *Calling AE Title* refers to the *AE Title* of the SCU sending the packet to the target. Some targets are configured to accept packets only from specific SCU with a pre-defined Calling AE Title. In this case, if the *Calling AE Title* does not match the one configured on the target, the request will be rejected.

**Type III. Target that accepts any** ***Called AE Title*** **or** ***Calling AE Title***

This type refers to a target that accepts the request regardless of the *Calling AE Title* or *Called AE title*. Targets of this type will accept any *Calling AE Title* or *Called AE Title*, regardless of this field.

**Type IV. Target that only accepts specific** ***Called AE Title*** **and** ***Calling AE Title***

This type, in contrast to TYPE-III, refers to a target that only accepts requests with specific *Calling AE Title* and *Called AE title* that the target is configured to accept. If both elements do not match, the target will reject the request.

Based on this classification, all experimental targets in our evaluation are organized as shown in Table 5.

### 6.1. Experiment Result

**Figure 13:**
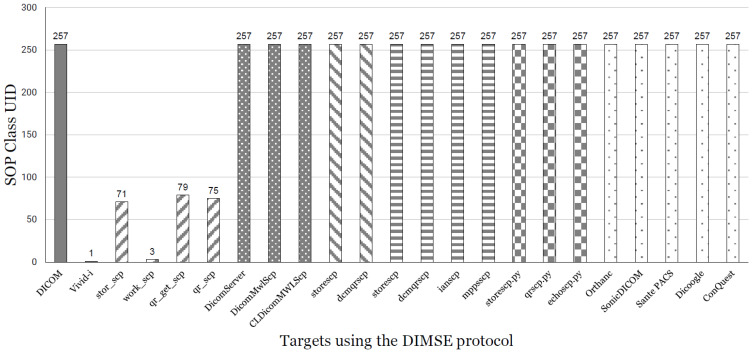
Number of *SOP Class UID* accepted by the target considering only the *PDU Type* field.

As stated in the *DICOM* standard, we have confirmed that there are two PDU types of response packets in the A-Associate stage. The two are acceptance (0 x2) and rejection (0 x3), which means that the request can either be accepted or rejected. Based on this information, we designed it to send all *SOP Class UID*s to a specific target, receive response packets, accept or reject them, and record them only in the case of acceptance. When conducting this experiment, the packet was captured and monitored through WireShark [46]. The result of experiments on the target using our framework *DIMScern* is shown in Figure 13.

The leftmost part is the number of *SOP Class UID*s defined in the *DICOM* standard. The other part is a list of targets that use the *DIMSE* protocol and indicates if the result of the response has the *PDU Type* as acceptance (0 x2). It is a graph of how many *SOP Class UID*s are accepted out of a total of 257. All 257 *SOP Class UID*s defined in the *DICOM* standard were accepted for targets except for *Vivid-i* and Merative targets. We have found that the *SOP Class UID* for C-ECHO, “1.2.840.10008.1.1”, is supported by all the targets we collected.

### 6.2. False Positive in Discerning Packet Responses

What can be inferred from the above experimental results is that all *DIMSE* operations should be possible for targets that accept 257 *SOP Class UID*s.

However, as a result of performing multiple *DIMSE* operations on the targets, the *DIMSE* commands (C-STORE, C-FIND, C-GET, etc.) were not executed in the P-Data phase. The framework we initially designed only considered the *PDU Type* field in the A-Associate packet to determine whether a particular *SOP Class UID* is accepted or rejected by the target. This baseline generated false positives, and we conducted a deeper analysis to determine that the *SOP Class UID* should not be determined solely by considering the *PDU Type* field. We have identified three possible cases that can occur in the response packet of the A-Associate.

**Figure 14:**
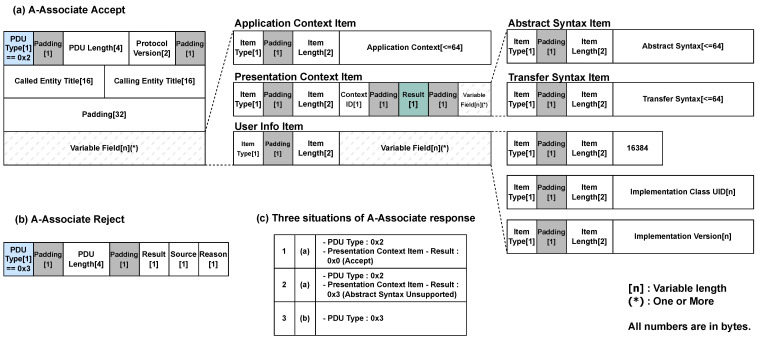
Possible situations in the A-Associate response.

Those three cases are presented in Figure 14. The response packet structure of A-Associate is largely divided into two. The structure of acceptance is *PDU Type* 0 x2 (Figure 14a), and the structure of rejection is *PDU Type* 0 x3 (Figure 14b). We split these two and tested whether the target would accept the *SOP Class UID*. When we performed the *DIMSE* command on a target that accepted 257, we found that not all commands were possible. When we analyzed the packet by field, we found that there are two situations in acceptance. This is the field of the *Presentation Context Result*. When SCU sends an *SOP Class UID* to a target, even if the target accepts it as a response, the *SOP Class UID* that the target accepts must be 0 x00 in the Result field, and if the field is 0 x03, it is an “Abstract Syntax Unsupported” *SOP Class UID* that the target does not accept. We summarized these three situations in Figure 14c and showed the *PDU Type* and the field of the *Presentation Context Result* for the situations that should be considered, including false positives.

**Figure 15:**
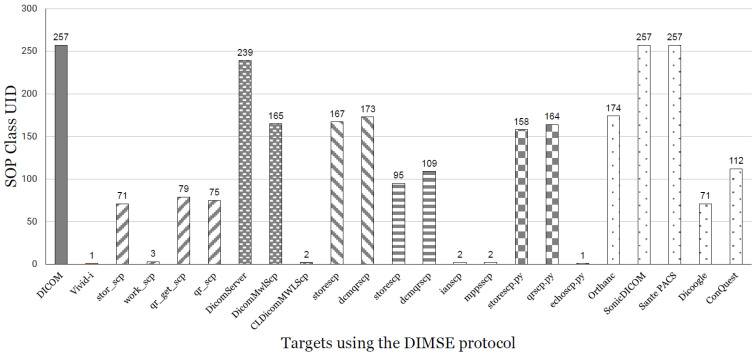
Number of *SOP Class UID* acceptance by the target, taking into account the *Presentation Context Result* Field.

Considering these three situations, the result of sending an *SOP Class UID* to the target with *DIMScern* and receiving a response is shown in Figure 15. To explain what is shown in the results, we can see that targets except SonicDICOM and Sante PACS accept only some of the *SOP Class UID*s defined in *DIMSE*. This means that each target accepts different *SOP Class UID*s, and more broadly, each target accepts different *DIMSE* commands. The reason why some targets accept fewer *SOP Class UID*s is that the target is a test program designed for a specific *DIMSE* command. So the test program provided by the Toolkit/Library accepts fewer *SOP Class UID*s than PACS/Server.

### 6.3. Information Leakage

**Figure 16:**
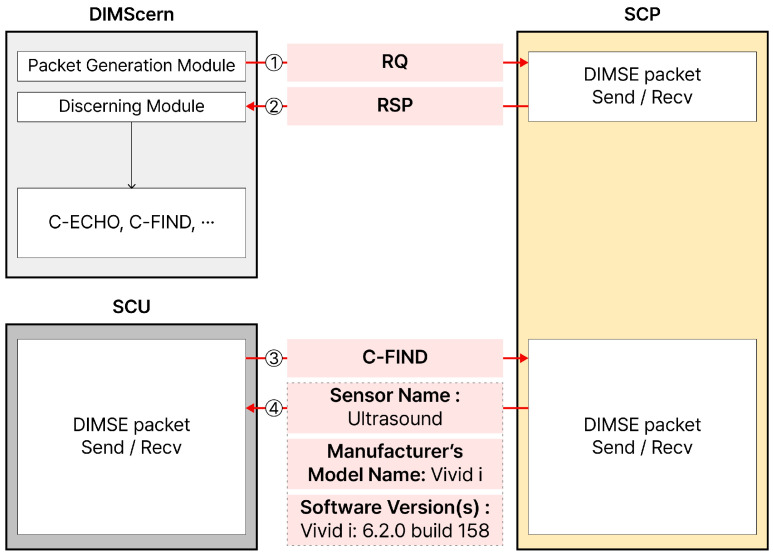
Relationship between *DIMScern* and sensor, device, and software information.

Through the framework *DIMScern*, we can classify possible commands for the target and then obtain sensor information, equipment information, or software version information. We conducted a simple experiment to verify whether C-FIND, a command that requests real medical data, can obtain sensor information, equipment information, or software version information for possible targets. This is shown in Figure 16.

Through *DIMScern*, a packet is sent to a target by including *SOP Class UID*s one by one from the list in the A-Associate request packet for a specific target (Figure 16①). After the target receives this packet, it sends a response packet to *DIMScern* (Figure 16②). *DIMScern* sends all *SOP Class UID*s in the list and records the *SOP Class UID* that the target has accepted. This allows it to distinguish which *DIMSE* commands are available for the target.

After that, if a target can be found via the C-FIND command, the SCU program is used to request sensor information, medical device information, or software version information for the target through the C-FIND command (Figure 16③). Then, the target that can be found via C-FIND sends the information that SCU wants, such as sensor information, medical device information, and software version information, in a response packet as a response (Figure 16④). We conducted an experiment to demonstrate how the C-FIND command can obtain information from a given target. For this experiment, we also need a dummy DICOM file, so we used the medical sensor (ultrasound) in the *Vivid-i* medical device to generate a DICOM file by measuring a random object. However, because *Vivid-i* does not support the C-FIND command, we assumed the Orthanc *DICOM* server, which supports the C-FIND command, and is considered as an SCP by copying the crafted DICOM file to the SCP ( Orthanc *DICOM* server).

**Figure 17:**
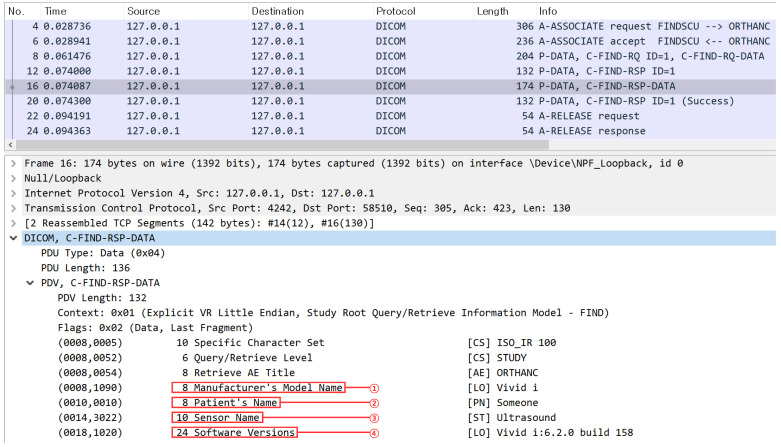
Packet capture that obtains *sensor information*, *medical device information*, and *software version* for targets that can be found via C-FIND.

After sending a crafted packet that contains the C-FIND command to SCP, we could observe that the SCP generated a response packet that contains sensitive information, such as the manufacturer’s model name (Figure 17①), the patient’s name (Figure 17②), the sensor name (Figure 17③), or the software version (Figure 17④), as shown in Figure 17.

### 6.4. Summary of Evaluation

We can remotely send an A-Associate request packet containing an *SOP Class UID* to a target using the *DIMSE* protocol via *DIMScern*, and evaluate its response to determine which *SOP Class UID* is accepted by the target and which *DIMSE* service is supported by the target system. Particularly, we correctly discern all medical services from 22 different *DIMSE* implementations.

By exploiting *DIMScern*, we can obtain a sensor identifier, medical device information, patient’s data, and software version from a target that supports the C-FIND command. This information is a critical factor for adversaries who plan more sophisticated security attacks. For example, medical device (or software) identifiers, along with their version information, can be exploited by adversaries to target specific security vulnerabilities (i.e., CVEs) within the systems, allowing them to tailor their attacks to known weaknesses.

## 7. Discussion

**Presentation Context Result Field Values:** In our framework, the *Discerning module* (shown in Figure 12) for A-Associate response packets falls into two categories: 0 x2 (acceptance) and 0 x3 (rejection). In the case where the *PDU Type* of the response packet is acceptance, we divided it again into two and included only the cases where the *Presentation Context Result* field is 0 x0 and 0 x3 to determine which *SOP Class UID* is accepted for the target. According to the *DICOM* standard, the possible values for the *Presentation Context Result* field are 0 x0 to 0 x4, which represent ’Acceptance’, ’User Rejection’, ’No Reason’, ’Abstract Syntax Unsupported’, and ’Transfer Syntax Unsupported’, respectively. For the targets we set, we did not find any values other than 0 x0 and 0 x3 in the *Presentation Context Result* field when the *PDU Type* is 0 x2 (acceptance). This means that when we later use our framework against a target that uses the new *DIMSE protocol*, if we observe a value other than 0 x0 or 0 x3 in that field, we will need to update the *Discerning module* (shown in Figure 12) of our framework.

**Framework Applicability in Security Contexts:** By examining various CVEs related to the *DIMSE* protocol, we can see that our framework has the potential to further contribute to security analysis.

*CVE-2015-8979* [18] vulnerability was discovered in the *storescp.exe* program, which is one of the test programs in the *DCMTK* [34]. If a crafted packet that has the C-STORE command is sent to the target program, a stack buffer overflow possibly occurs, resulting in a denial of service.

*CVE-2019-5090* [19] vulnerability was found in a packet processing library, Leadtools [36]. An adversary can extract sensitive information through out-of-bound memory accesses caused by a crafted C-GET command packet.

*CVE-2019-5093* [20] vulnerability was found in a packet processing library, Leadtools [36] as well, where a crafted C-FIND command packet can cause an integer overflow and heap corruption.

*CVE-2024-34508* [21] and *CVE-2024-34509* [22] were introduced recently in 2024 and they target *DCMTK* [34]’s test programs and cause segmentations faults via a crafted C-STORE command packet.

With the *DIMScern* framework, an adversary can determine which *DIMSE* commands are supported by a given target and generate the crafted packets accordingly to the trigger security vulnerabilities that are listed above.

**Security Threats of Unprotected** ***DIMSE*** **Protocol Without Authentication Procedures:** The underlying reason our framework functions effectively and enables information leakage through *DIMSE* commmands is the absence of a built-in authentication mechanism within the *DIMSE* protocol. Desjardins et al. [29] revealed that over 1100 unprotected *DICOM* servers were discovered using shodan [30]. Among the *DICOM* servers categorized as Type-III, as defined in Section 6, our framework is capable of identifying all supported *DIMSE* commands, which can subsequently be exploited to enable different security attacks.

**Applicability of Our Framework in Medical Healthcare IT Systems:** Security analysts and professionals can utilize our framework in two primary ways. This framework can be applied during the security evaluation process of medical devices and healthcare systems in hospitals to identify potential pathways through which medical data could be exposed externally. By using our framework, *DIMScern*, it is possible to identify which *DIMSE* commands can be executed from external sources, thereby enabling the proactive detection of potential medical data leakage pathways. Another way is for developers at manufacturers or companies that design products or systems. Through *DIMScern*, developers can verify which commands are implemented to be accessible, allowing them to identify and address information that could be exposed externally in advance. This allows for the formulation of effective countermeasures. From this perspective, our framework is expected to contribute significantly to proactively identifying security issues and establishing robust security measures in real-world medical systems.

## 8. Conclusions

Security concerns on healthcare IT systems have escalated within the cybersecurity domain as the systems manage sensitive biomedical imaging data and expand their connectivity across networks. Although numerous security threats to medical systems with remote network access have been extensively studied, the initial phase of most security attacks involves gathering critical information about the remote system. To address this challenge to collect information from a remote healthcare IT system, we propose a framework, *DIMScern*, to demonstrate how it can be used to identify the medical services supported by a target medical system. Furthermore, we explain how the results from the *DIMScern* framework can be leveraged to expose sensitive information (e.g., biomedical imaging data, software version details) and facilitate various security exploits.

## Figures and Tables

**Table 1:** The highest-level packet field of the A-Associate stage.

Field	Description
PDU Type	This field indicates the type of *DIMSE* packet. The types include A-Associate request/acceptance/rejection.
Padding	Padding usually contains 0 x00. If the padding field has a length of more than 1 byte, all consist of 0 x00.
Protocol Version	In *DIMSE*, this field is currently restricted to the value 0 x0001.
Called AE Title	If there is a sender and a receiver, it refers to the recipient’s AE Title. An Application Entity (AE) Title is a field for indicating the identity of each application.
Calling AE Title	This refers to the sender’s AE Title.
Variable Field	This field has a variable length and contains other subfields.

**Table 2:** The highest-level packet field of the P-Data stage.

Field	Description
PDU Type	This field indicates the type of *DIMSE* packet. The types include P-Data request/response.
Padding	Padding usually contains 0 x00.
Variable Field	This field has a variable length and contains other subfields.

**Table 3:** The Values Contained in the *PDV DICOM Message* Field.

Tag	Length	Tag Name	Value
(0000, 0000)	4	Command Group Length	56
(0000, 0002)	18	Affected SOP Class UID	1.2.840.10008.1.1
(0000, 0100)	2	Command Field	C-ECHO-RQ
(0000, 0110)	2	Message ID	1
(0000, 0800)	2	Command Data Set Type	257

**Table 4:** Fields filled with default values for A-Associate request.

Field	Value
Protocol Version	1
Called Entity Title	DIMSESCP
Calling Entity Title	DIMScern
Application Context	1.2.840.10008.3.1.1.1
Transfer Syntax	1.2.840.10008.1.2

**Table 5:** Information about targets using the *DIMSE* protocol.

Target	Description	Test Program	Type
Vivid-i [33]	Ultrasonic medical device	-	Type-III
Merative [35]	DICOM Toolkit/CLI Test program	stor_scp.exe	TYPE-I
		work_scp.exe	Type-I
		qr_get_scp.exe	Type-I
		qr_scp.exe	Type-I
Leadtools [36]	DICOM SDK Library/GUI Test program	DicomServerDemo_Original.exe	Type-II
		DicomMwlScpDemo_Original.exe	Type-IV
		CLDicomMWLScp_Original.exe	Type-IV
DCMTK [34]	DICOM Toolkit & Library/CLI Test program	storescp.exe	Type-III
		dcmqrscp.exe	Type-I
dcm4che [37]	DICOM Toolkit & Library/CLI Test program	storescp.exe	Type-I
		dcmqrscp.exe	Type-I
		ianscp.exe	Type-I
		mppsscp.exe	Type-I
pynetdicom [38]	Python Library/CLI Test program	storescp.py	Type-III
		qrscp.py	Type-III
		echoscp.py	Type-III
Orthanc [50]	DICOM PACS/GUI program	-	Type-III
SonicDICOM [51]	DICOM PACS/GUI program	-	Type-III
Sante [52]	DICOM PACS Server/GUI program	-	Type-III
Dicoogle [53]	DICOM PACS/CLI program	-	Type-I
ConQuest [54]	DICOM Server/GUI program	-	Type-III

## Data Availability

The DICOM file shown in Figure 2 is derived from the “TCGA-LGG” dataset, specifically file “99-190.dcm” (TCGA-CS-5396), originally created and provided by The Cancer Imaging Archive (TCIA) and accessible via CyVerse Data Commons [23] under the Creative Commons Attribution 3.0 Unported License (https://creativecommons.org/licenses/by/3.0/. accessed on 22 October 2024). Note that the original data were not modified; however, the *Data Set* and *Payload* in Figure 2 represent parts of the original data displayed through a screen capture, and we use the same DICOM file to show the *DICOM binary file* part in Figure 2. The captured packet files used in Figure 11 and Figure 17, the crafted DICOM file used in Figure 17, and the code for the framework presented in this paper were all created by us and they are publicly available in our Github repository (https://github.com/CASO-Lab/DIMScern.git. accessed on 22 October 2024).

## Abbreviations

The following abbreviations are used in this manuscript:

IT	Information Technology
S/W	Software
DICOM	Digital Imaging Communications in Medicine
DIMSE	DICOM Message Service Element
CVE	Common Vulnerabilities and Exposures
ACR	American College of Radiology
NEMA	National Electrical Manufacturers Association
PACS	Picture Archiving and Communication Systems
SCU	Service Class User
SCP	Service Class Provider
PDU	Protocol Data Unit
UID	Unique Identifiers
SOP	Service-Object Pair
VR	Value Representation
PDV	Presentation Data Values
ECG	Electrocardiogram

LAN	Local Area Network
CPU	Central Processing Unit
HDD	Hard Disk Drive
RAM	Random Access Memory
VDI	Virtualbox Disk Image
PID	Process Identifier
TCP	Transmission Control Protocol
AE	Application Entity
CT	Computed Tomography
API	Application Programming Interface
IP	Internet Protocol
